# Dose–Response Relationship Between Serum 25(OH)D and the Risk of Abnormal Glycemic Status in Chinese Older Adults

**DOI:** 10.3390/metabo14110579

**Published:** 2024-10-26

**Authors:** Yuting Li, Jing Chen, Qingqing Man, Rui Wang, Deqian Mao, Min Li, Jian Zhang, Yichun Hu, Lichen Yang

**Affiliations:** Key Laboratory of Public Nutrition and Health, National Health Commission of the People’s Republic of China, National Institute for Nutrition and Health, Chinese Center for Disease Control and Prevention, 29 Nanwei Road, Beijing 100050, China; lytting@yeah.net (Y.L.); chenjing@ninh.chinacdc.cn (J.C.); manqq@ninh.chinacdc.cn (Q.M.); wangrui@ninh.chinacdc.cn (R.W.); maodq@ninh.chinacdc.cn (D.M.); limin@ninh.chinacdc.cn (M.L.); zhangjian@ninh.chinacdc.cn (J.Z.)

**Keywords:** 25-hydroxyvitamin D, type 2 diabetes mellitus, prediabetes, abnormal glycemic status, older adults, restricted cubic spline regression, dose–response analysis

## Abstract

Background/Objectives: The relationship between serum 25-hydroxyvitaminD (25(OH)D) concentration and the risk of abnormal glycemic status remains contradictory. Whether sufficient 25(OH)D plays a role in reducing the risk of diabetes and prediabetes is debatable. Its dose–response relationship and the optimal 25(OH)D threshold are not clear. This study investigated the relationship between serum 25(OH)D and the risk of abnormal glycemic status to explore the optimal cut-off value. Methods: This study included 5856 Chinese older adults aged 60 years and above from the China Adult Chronic Disease and Nutrition Survey (CACDNS 2015–2017). Serum 25(OH)D concentration was measured via an enzyme-linked immuosorbent assay. Abnormal glycemic status in the present study includes diabetes and prediabetes. Statistical methods such as a locally weighted regression and smoothing scatterplot (LOESS), restricted cubic spline regression (RCS) and binary and trend logistic regression were used to explore the dose–response relationship and the optimal 25(OH)D threshold. Results: The concentration of 25(OH)D was nonlinearly related to the levels of FPG and HbA_1C_ in the LOESS curves. The nonlinear relation between 25(OH)D and the risk of diabetes and prediabetes was illustrated in the RCS curves and the optimal 25(OH)D threshold beneficial for diabetes was preliminarily explored to be 29.06 ng/mL, but no threshold for prediabetes was found. The dose–response results showed that for each 1 ng/mL increase in 25(OH)D, the risk of the fasting glucose type of diabetes reduced by 2.1%, the risk of the abnormal HbA_1C_ type of diabetes reduced by 2.2% and the risk of the mixed type of diabetes reduced by 1.7%, whereas a dose–response relationship was not found for prediabetes. Conclusions: Higher serum 25(OH)D concentrations in Chinese older adults were associated with a reduced risk of glycemic abnormalities. The optimal 25(OH)D cut-off value was sufficiently beneficial if the diseased diabetes risk was 29.06 ng/mL, but no threshold was found for prediabetes.

## 1. Introduction

Type 2 diabetes mellitus (T2DM) has been a global health concern due to its high prevalence and multiple complications. Prediabetes (pre-DM) is an intermediate, reversible state of abnormal glycemic status between diabetics and normal glycemic individuals, in which people already have elevated levels of fasting plasma glucose (FPG) and glycosylated hemoglobin (HbA_1C_) due to insulin resistance and pancreatic β-cell dysfunction in the early stages of the disease [1]. The International Diabetes Federation (IDF) reported that there were 536.6 million T2DM adults aged 20~79 worldwide in 2021, with China topping the list with 140.9 million [2]. The number of people with T2DM in China is predicted to increase to 174.4 million by 2045 and the number of people with T2DM globally will increase to 783.2 million. It is especially noteworthy that the prevalence of glycemic abnormalities is even more serious in the elderly Chinese population. For example, as of 2018, the prevalence of T2DM was 23.9% in older adults aged 60~69 years and 27.3% in older adults aged 70 years and older, whereas the prevalence of pre-DM in older adults aged 60~69 years was 47.6%, and 48.9% in those aged 70 years and above [3]. In addition, the economic burden of T2DM is predicted to grow faster than China’s economic growth during 2020–2030 [4]. Abnormal glycemic status can also lead to a higher risk for certain kinds of chronic diseases such as nephropathy, retinopathy [5], metabolic syndrome and even cardiovascular disease [6], stroke and all-cause mortality [7], which affects the health and well-being of the elderly to a great extent.

Although the physiologic role of vitamin D has attracted widespread attention, its role in T2DM has not been elucidated and the debate over whether more sufficient vitamin D is beneficial for T2DM remains contradictory [8,9]. Only a handful of observational studies have investigated the association between vitamin D and the incidence of T2DM, with contradicting results from western populations. For instance, the results of a momentous meta-analysis of 21 prospective studies in 2013 showed a 38% reduction in the risk of T2DM in the highest category of 25(OH)D compared to the lowest category, and yielded a linear inverse relationship [10]. A meta-analysis of observational and case–control studies in 2022 showed that the highest compared to the lowest levels of vitamin D were, respectively, associated with a 35%, 51% and 30% decrease in the risk of T2DM, pre-DM, combined T2DM and pre-DM [11]. However, results from the Irish Longitudinal Study on Ageing showed that vitamin D deficiency (<12 ng/mL) increases the risk of pre-DM by 62% in people aged 50 years and older, but has no effect on T2DM, which may be attributable to the limited power of low T2DM incidents [12]. No consensus has been reached in RCT studies. For example, the vitamin D and type 2 diabetes (D2d) trial in the US population showed via subgroup analysis that vitamin D supplementation reduced the risk of T2DM only in vitamin D-deficient normal-weight adults [13]. A meta-analysis of 8 RCT trials found that vitamin D supplementation increased the proportion of pre-diabetic return to normal and lowered the risk of T2DM [14], whereas another meta-analysis based on 35 RCT trials showed that vitamin D_3_ supplementation had no effect on the maintenance of glucose homeostasis or the prevention of T2DM [15].

To date, there is no worldwide consensus on the appropriate cut-off value of vitamin D. Guidelines for interpreting 25(OH)D concentration are mainly derived from bone metabolism studies [16]; the Institute of Medicine (IOM) [17] suggests that 20 ng/mL can be regarded as sufficient for the general population, whereas the Endocrine Society (ES) 2024 [18] discards 30 ng/mL as sufficient, suggesting that dosages should be tailored to specific populations or specific diseases statuses, such as in children, the pregnant, adults over 75 years of age and individuals with pre-DM at high risk for T2DM. Specific supplemental doses should be based on the vitamin D concentrations to be protective against specified diseases statuses in clinical trials. Obtaining sufficient serum 25(OH)D levels for the prevention of T2DM is essential for the development of proposed intakes for preventing non-communicable chronic diseases (PI-NCD).

Data from the China Adult Chronic Disease and Nutrition Survey (CACDNS, 2015–2017) showed that nearly 58.27% of Chinese older adults aged 60 years or older had vitamin D deficiency and insufficiency [19]. Few studies based on large nationally representative data of Chinese older adults have investigated the dose–response relationship between vitamin D nutritional status and abnormal glycemic status. The optimal 25(OH)D cut-off value as sufficiently beneficial for diseased diabetes has not been fully explored in elderly populations and high-risk pre-diabetics.

Given that the promising effects of 25(OH)D on T2DM are ambiguous, and given severe vitamin D deficiency and the high prevalence of T2DM and pre-DM in the Chinese older adult population, we aimed to investigate the relationship between serum 25(OH)D and glycemic abnormalities and attempted to find the optimal serum 25(OH)D cut-off value for the prevention of T2DM in this representative large-scale study. The results of this study can also be used as basic information and reference for further research.

## 2. Materials and Methods

### 2.1. Study Population and Sampling

The analysis was based on data from the China Adult Chronic Disease and Nutrition Survey (CACDNS 2015–2017) conducted by the Chinese Center for Disease and Control Prevention (China CDC). This survey covered the county (district) level administrative units (counties, county-level cities and districts) in all 31 provinces in mainland China. A complex stratified cluster random selection procedure was used to select participants and establish a biospecimen bank. The participants who were ≥60 years of age were recruited with detailed socio-demographic and chronic disease information. The quality of the blood samples was assured and informed consent forms were signed before the start of the survey. Assuming that the prevalence of diabetes in the Chinese population over 60 years of age is 20.2% according to a study by Wang et al. [20], the sample size for each stratification factor was estimated at 581 subjects. Considering gender and age group (60~, 65~, 70~, 75~, 80~), there were 10 strata and a total of 5810 individuals were randomly sampled from the survey based on the distribution of 289 monitoring sites in China. A total of 5856 participants aged 60 years and older with complete sociodemographic information and blood samples free of hemolysis were recruited. The study was approved by the Ethical Review Committee of National Institute for Nutrition and Health (NINH), Chinese Center for Disease Control and Prevention (No. 201519-B, approved on 15 June 2015).

### 2.2. Data Collection and Definition of Variables

The CDC established a national working group to conduct this survey. Interviews and anthropometric measurements were conducted, and venous blood was collected. A standardized questionnaire was used to collect basic information on age, gender, ethnicity, region type, district, education level, marital status, smoking, alcohol consumption, physical activity and disease status. Height and weight were measured via a uniform method. Latitude was taken from the Baidu map (https://map.baidu.com, accessed on 28 July 2024). All the information was collected and entered into the CACDNS 2015–2017 system platform. Education level was categorized as primary (elementary and below), medium (junior middle school/senior high school) and advanced (junior college and above). Spring (March to May), summer (June to August), autumn (September to November) and winter (December to February) were used to classify seasons. Other variables were defined with reference to the previous literature [19]. High-intensity activity was defined as activities that require considerable physical effort or may cause a significant increase in respiration or heart rate, such as long-distance running, swimming, etc. for at least ten minutes [21]. Cigarette smoker referred to current or former smokers. The participants were categorized as alcohol consumers if they had a history of alcohol consumption in the past year. Body mass index (BMI) was calculated as weight (kg) divided by the square of height (m^2^).

### 2.3. Laboratory Measurements

Fasting venous blood (10 mL) was drawn from each participant and placed in EDTA-K2 anticoagulated and separated gel blood collection tubes, respectively. Plasma and serum samples were obtained via centrifugation at 3000 rpm for 15 min and stored at −80 °C for analysis. Biochemical tests including fasting plasma glucose (FPG), total cholesterol (TC), triglycerides (TG), high-density lipoprotein cholesterol (HDL-c), low-density lipoprotein cholesterol (LDL-c) and plasma uric acid (UA), were measured by a fully automated biochemical analyzer (Hitachi 7600, Tokyo, Japan); glycosylated hemoglobin (HbA_1C_) was determined via a high-performance liquid chromatography (HPLC) method (Trinity Biotech Premier Hb9210, Dublin, Ireland) and hemoglobin (Hb) was detected spectrophotometrically using the high cyanide–iron method. The indicators all passed quality control and the coefficients of variation (CV) between and within the batches were less than 5% for UA, less than 10% and 3% for FPG and less than 2% for all the others. In this study, serum 25(OH)D concentration was detected using an ELISA kit (Immune Diagnostic System Ltd., Boldon, UK). Quality control was performed using the kit’s own quality control and 10% parallel samples; the CVs for the kit at high and low values were 6.69% and 6.94% with deviations of 1.26% and 2.72%, respectively, and the CV for the parallel samples was 4.86%.

### 2.4. Definition of Diabetes and Prediabetes

The diagnosis of T2DM and pre-DM was based on the 2024 edition of the American Diabetes Association (ADA) criteria, Diagnosis and Classification of Diabetes: Standards of Care in Diabetes [22].

T2DM was diagnosed when any of the following were met: (1) fasting glucose type of T2DM: FPG ≥ 7.00 mmol/L; (2) abnormal HbA_1C_ type of T2DM: HbA_1C_ ≥ 6.5% (≥48 mmol/mol); (3) mixed type of T2DM: consistent with (1) or (2) stated above. Pre-DM was diagnosed when any of the following were met: (1) fasting glucose type of pre-DM: FPG in the range of 5.60~6.99 mmol/L; (2) abnormal HbA_1C_ type of pre-DM: HbA_1C_ in the range of 5.7~6.4% (39~47 mmol/mol); (3) mixed type of pre-DM: consistent with (1) or (2).

### 2.5. Statistical Analysis

Descriptive statistics for the study population were obtained by calculating the mean ± standard deviation (SD) or median (IQR) for continuous variables and count (%) for categorical variables. Kruskal–Wallis’s test was used to compare the differences between the groups. A chi-square test was used for the comparison of the ratio or proportion. Locally a weighted regression and smoothing scatterplot (LOESS) was used to explore the relationship between numerical variables such as 25(OH)D, FPG and HbA_1C_. Before the use of LOESS, multiple linear regression was used to find the confounders of variables and then a generalized additive model (GAM) was used to adjust those confounders [23]. To explore the optimal 25(OH)D threshold and the dose–response relationship between 25(OH)D concentration and the risk of T2DM and pre-DM, restricted cubic spline regression (RCS) with 5 knots (5th, 25th, 50th, 75th and 95th) was used. Binary logistic regression was used to analyze the relationship between 25(OH)D levels and the risk of T2DM and pre-DM. Trend binary regression was used to explore the dose–response relationship between 25(OH)D concentration and disease risk. Analyses were performed with IBM SPSS version 23.0 (IBM Corporation, Armonk, NY, USA) and SAS version 9.4 (SAS Institute, Cary, NC, USA). A two-sided *p*-value < 0.05 was considered statistically significant.

## 3. Results

### 3.1. Basic Characteristics

A total of 5856 participants were included in this study with the median age of 73.14 years; 50.82% were men, of which 59.02% were from urban areas and 40.98% were from rural areas. The percentage of participants from eastern, central and western China were 34.29%, 30.99% and 34.72%, respectively (Table 1).

### 3.2. Vitamin D Status and Clinical Characteristics Based on Different Glycemic Status

The clinical characteristics of different glycemic status groups are shown in Table 2. The median concentration of 25(OH)D in the T2DM group (17.31 ng/mL) was significantly lower than that in the pre-DM (18.90 ng/mL) and normal (18.73 ng/mL) groups and no significant difference between the latter two groups was found. The median of the BMI, waist circumference, heart rate, TG, FPG and HbA_1C_ were lowest in the normal group, followed by the pre-DM group and the T2DM group (*p* < 0.001). The median of LDL-c and Hb were higher in the T2DM and pre-DM groups than in the normal group, but there was no statistically significant difference between the T2DM and pre-DM group. The median of HDL-c was higher in the normal group than in the T2DM and pre-DM groups (Table 2).

### 3.3. Correlation Coefficient Between Variables

**Figure 1:**
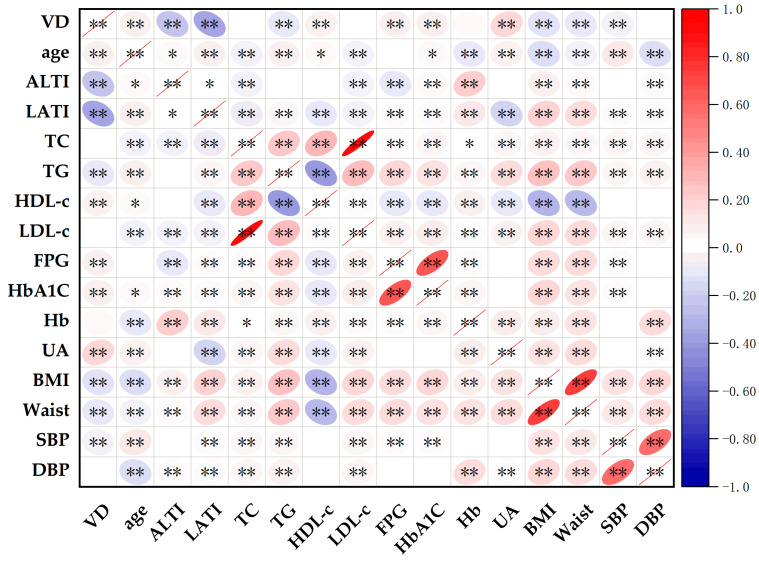
Pearson correlation coefficient matrix heatmap. Color labeling: red color indicates that the correlation coefficient is positive; blue color indicates that the correlation coefficient is negative. The self-correlation coefficient in the figure can be ignored and has been crossed out with red lines. Significance labeling: * *p* ≤ 0.05; ** *p* ≤ 0.01. Abbreviation: VD, 25-hydroxyvitamin D; ALTI, altitude; LATI, latitude; TC, total cholesterol; TG, triglycerides; HDL-c, high-density lipoprotein cholesterol; LDL-c, low-density lipoprotein cholesterol; FPG, fasting plasma glucose; HbA_1C_, glycated hemoglobin; Hb, hemoglobin; UA, uric acid; BMI, body mass index; SBP, systolic blood pressure; DBP, diastolic blood pressure.

As shown in Figure 1, it was revealed that the 25(OH)D concentration was negatively correlated with latitude, altitude, BMI, TG, waist, SBP, HbA_1C_, age, FPG and heart rate and, conversely, 25(OH)D was positively correlated with UA and HDL-c. Glycemic trait indicators, such as FPG, were negatively correlated with 25(OH)D and HDL-c, and altitude and was positively correlated with HbA_1C_, TG, BMI, waist, heart rate, LDL-c, TC, SBP, latitude, and Hb. As for HbA_1C_, it was negatively correlated with 25(OH)D, HDL-c, altitude, and age and was positively correlated with FPG, BMI, waist, TG, LDL-c, Hb, TC, heart rate, SBP, and latitude. All the correlation coefficients were shown in Supplementary Table S1 (Pearson correlation coefficient between variables).

### 3.4. Nonlinear Relationship Between Vitamin D Concentration and Glycemic Traits Indicators

**Figure 2:**
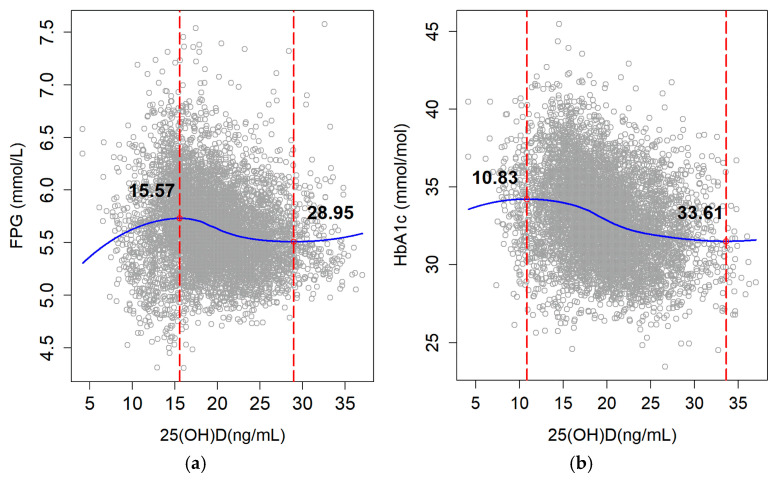
25(OH)D thresholds for glycemic traits by LOESS analysis. (**a**) Association between serum 25(OH)D concentration and FPG level. (**b**) Association between serum 25(OH)D concentration and HbA_1C_ level. In (**a**,**b**), serum 25(OH)D was adjusted for age, gender, education level, region type, district, season, altitude, latitude, BMI, drinking, TG, Hb, UA. In (**a**), FPG was adjusted for age, region type, district, altitude, BMI, smoking, TG, Hb, UA. In (**b**), HbA_1C_ was adjusted for ethnicity, region type, district, BMI, TC, TG, HDL-c, Hb, UA. Abbreviations: 25(OH)D, 25-hydroxyvitamin D; BMI, body mass index; TC, total cholesterol; TG, triglycerides; HDL-c, high-density lipoprotein cholesterol; LDL-c, low-density lipoprotein cholesterol; FPG, fasting plasma glucose; HbA_1C_, glycated hemoglobin; Hb, hemoglobin; UA, uric acid.

The multiple linear regression of serum 25(OH)D concentration and glycemic traits such as FPG and HbA_1C_ was carried out and the partial correlation coefficient of each variable was shown in Supplementary Table S2 (Partial correlation coefficient of serum 25(OH)D concentration and glycemic traits by multiple linear regression). After the adjustment of relevant confounding factors, a nonlinear relationship was found between the 25(OH)D concentration and FPG level in the LOESS curve (Figure 2a) and the 25(OH)D concentration was 28.95 ng/mL when the FPG reached a plateau. The 25(OH)D concentration was also nonlinearly related to the level of HbA_1C_ (Figure 2b), with the elevation of the 25(OH)D concentration and the level of HbA_1C_ decreasing gradually, and as HbA_1C_ reached a plateau, the 25(OH)D concentration was 33.61 ng/mL.

### 3.5. Vitamin D Cut-Off Value for the Prevention of Abnormal Glycemic Status

**Figure 3:**
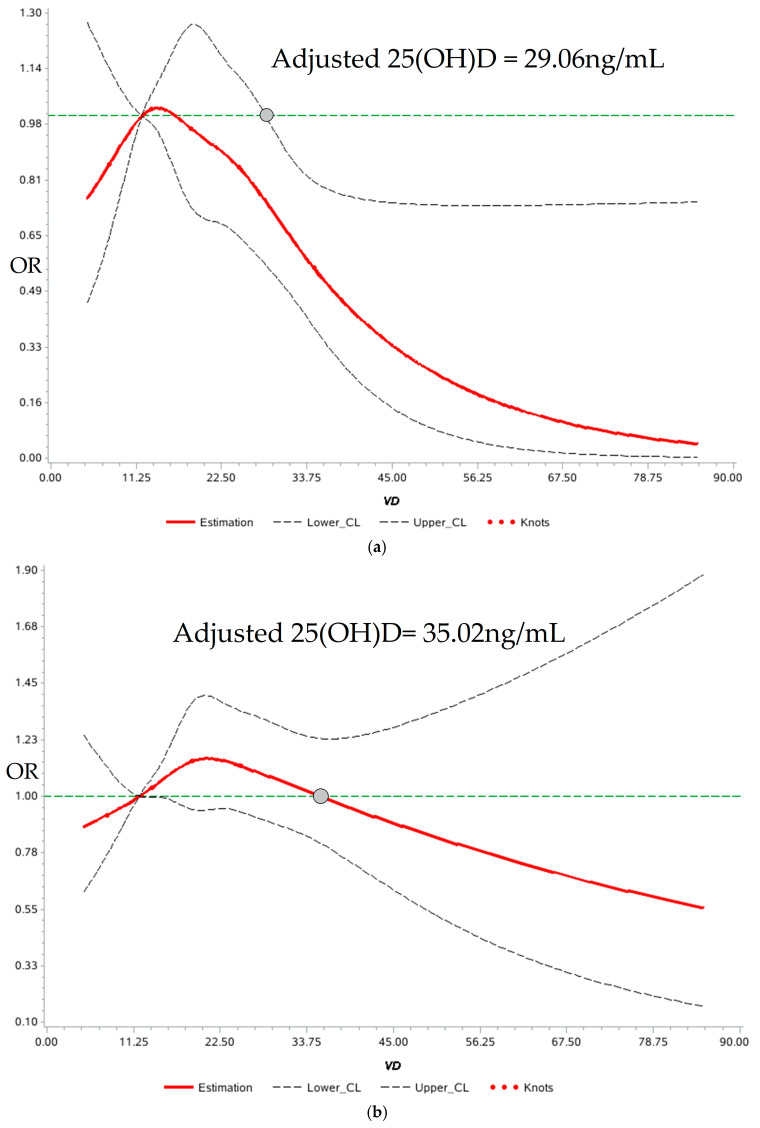
25(OH)D cut-off value for abnormal glycemic status via RCS analysis. (**a**) Association between serum 25(OH)D concentration and diabetes. (**b**) Association between serum 25(OH)D concentration and pre-diabetes. The green horizontal dashed line indicates an OR value of 1. Serum 25(OH)D was adjusted for age, gender, ethnicity, education level, region type, district, season, altitude, latitude, BMI, physical activity, smoking and drinking, TC, TG, HDL-c and UA. Abbreviations: 25(OH)D, 25-hydroxyvitamin D; BMI, body mass index; TC, total cholesterol; TG, triglycerides; HDL-c, high-density lipoprotein cholesterol; UA, uric acid.

Figure 3a showed that the 25(OH)D concentration was nonlinearly correlated with the risk of T2DM in the RCS curve using 12 ng/mL as a reference (*p*_overall_ < 0.05, *p*_nonlinearity_ < 0.05). The thresholds were fitted using different statistical methods. First, relevant confounders were adjusted in the RCS with reference to Table 3 (Model 3) and the 25(OH)D concentration of 29.06 ng/mL was chosen as the cut-off value that was favorable for the prevention of T2DM (OR = 0.728, 95%CI: 0.546~0.970), above which the beneficial effect continued to increase as the OR value decreased. Additionally, binary logistic regression validation showed a reduced risk in T2DM development when the 25(OH)D concentration went up to 29.06 ng/mL in all the models, compared with the deficiency group (<12 ng/mL) (Table 3). After the adjustment of the sociodemographic factors, lifestyle factors and biochemical indicators (Model 3), the beneficial effect remained for the fasting glucose type of T2DM (OR = 0.518, 95%CI: 0.332~0.808, *p* = 0.004), the abnormal HbA_1C_ type of T2DM (HbA_1C_ ≥ 6.5%, OR = 0.561, 95%CI: 0.327~0.961, *p* = 0.035) and the mixed type of T2DM (OR = 0.649, 95%CI: 0.436~0.967, *p* = 0.033).

Figure 3b showed that the 25(OH)D concentration was nonlinearly correlated with the risk of pre-DM (*p*_overall_ < 0.05, *p*_nonlinearity_ < 0.05). Using the deficient status (<12 ng/mL) as a reference in RCS, no statistically significant cut-off value for pre-DM was found, with the OR value decreasing below 1 level when the 25(OH)D reached 35.02 ng/mL and the upper limit of OR remained above 1 as the 25(OH)D level increased. The cut-off value of three types of pre-DM were also not found in binary logistic regression even when 25(OH)D concentration went up to 35.02 ng/mL, compared to the deficiency group (<12 ng/mL) (Table 4).

As shown in Table 3, a negative dose–response relationship between the 25(OH)D concentration and the risk of developing T2DM was demonstrated in the trend regression. The dose–response results showed that for each 1 ng/mL increase in the 25(OH)D, the risk of the fasting glucose type of T2DM reduced by 2.1%, the risk of the abnormal HbA_1C_ type of T2DM reduced by 2.2% and the risk of the mixed type of T2DM reduced by 1.7%. But, there was no significant dose–response relationship between 25(OH)D concentration and the risk of pre-DM in trend regression (Table 4). Although the OR values of the abnormal HbA_1C_ type of pre-DM was lower than 1 in three models, none of them were significant (*p* > 0.05) (Table 4).

## 4. Discussion

Vitamin D has received much attention for its role in extra-skeletal health, especially for one of the major metabolic diseases, T2DM. However, the relationship between 25(OH)D concentration and T2DM has not been reported in a large representative sample of the Chinese elderly population. In the present study, we found that the concentration of 25(OH)D was nonlinearly related to the levels of FPG and HbA_1C_ in the LOESS curve. Then, we found a nonlinear relationship between the 25(OH)D concentration and the risk of T2DM in the RCS curves, with a “parabola-type” shape of elevated risk at low 25(OH)D concentrations and a reduced risk at higher concentrations. Yet, a 2013 meta-analysis of 21 prospective studies showed a monotonous linear inverse relationship [10]. There are fewer studies reflecting these morphological correlations to clearly illustrate this relationship; two recent cohort studies [24,25] conducted in eastern and western China were consistent with our results, both finding a nonlinear relationship between 25(OH)D concentration and T2DM via the RCS curve.

Many studies have focused on the dose–response relationship between 25(OH)D and glycemic abnormalities. Our study showed that for every 1 ng/mL increase in the 25(OH)D concentration, the likelihood of T2DM decreased by 1.7~2.2% for all three of the diagnosis criteria. Consistent with our findings, a meta-analysis of prospective studies in 2013 showed a 4% reduction in the likelihood of T2DM for every 4 ng/mL increase in 25(OH)D [10]. The result of the Tromsø study in 2023 found that for every 5 nmol/L increase in 25(OH)D concentration, the risk of T2DM was reduced by 21% in women and 10% in men in the 2001 transect [26]. In line with our findings, few Mendelian randomization (MR) studies have shown a causal and dose–effect relationship between 25(OH)D levels and T2DM. For example, the results of Lu et al.’s 2018 meta-analysis [27] based on the Chinese CKB database and European population showed a 14% reduction in the risk of T2DM for every 25 nmol/L increase in genetically predicted 25(OH)D levels, using vitamin D synthesis-associated SNP (rs12785878, rs10741657) as an instrumental variable; conflicting results were shown when another two SNP were added and it became non-significant, probably because the SNPs were pleiotropic. Another European population-based study conducted by Yuan et al. in 2019 [28] showed that genetically predicted 25(OH)D were negatively correlated with T2DM and, per standard deviation (SD, 0.33 ln-nmol/L), the increase in the 25(OH)D level reduced the risk of T2DM by 6% and 10%, respectively, using seven and three SNPs as instrumental variables. In addition, a bidirectional MR study based on the South Asian and European population [29] in 2021 found a 5% increase in the risk of T2DM for every 4.2 nmol/L decrease in the 25(OH)D concentration using the 25(OH)D synthesis SNP (rs12785878) as an instrumental variable. However, no significant dose–response relationship was found for pre-DM in our study, probably because its vitamin D nutritional and glycemic statuses was not significantly different from that of the normal. Similar to our study, a 2022 meta-analysis of prospective studies [11] showed that for every 10 ng/mL increase in circulating 25(OH)D, there was a significant 12% reduction in the risk of T2DM and an 11% reduction in the risk of combined T2DM and pre-DM, whereas there was a 19% reduction in the risk of pre-DM, but it was not significant (95% CI: 0.60~1.09).

To date, there is no consensus on the optimal 25(OH)D thresholds for T2DM, and relevant studies are limited. In this study, we preliminarily explored a 25(OH)D level of 29.06 ng/mL as a promising effective threshold for the prevention of T2DM in Chinese older adults. It is worth noting that the threshold had beneficial effects on all three types of T2DM. A meta-analysis of prospective studies showed that a higher serum 25(OH)D level was monotonically associated with a lower risk of T2DM, suggesting that there is no clear threshold for benefit [10]. Other studies have confirmed vitamin D and T2DM in a nonlinear inverse relationship with different thresholds. For example, a study of adults over 18 years of age in eastern China found a nonlinear relationship between 25(OH)D concentration and T2DM, but no statistically significant threshold was found [24]; another study of adults aged 40~75 years in western China found a parabola-type relationship which was similar to ours, but due to the upper limit of its 95%CI, was consistently greater than 1; no threshold was obtained [25]. A study of the genetically predicted 25(OH)D threshold for T2DM in European adults aged 35~65 years found nonlinearity with a threshold of 18 ng/mL (45 nmol/L) [30]. In a medium-sized cross-sectional study of overweight, obese postmenopausal women in Maryland, the threshold for glycemic traits consisted of glucose homeostasis and insulin resistance, and was 21~26 ng/mL [31]. Our older adults had a threshold of 29.06 ng/mL, which was slightly higher than the above findings of the western study. So far, the PI-NCD value for 25(OH)D in the elderly has not been tailored for T2DM, especially for the Chinese population, and we believe that our findings can provide basic data for public health maintenance, especially for older adults at a high risk of severe glycemic abnormalities and vitamin D hypovitaminosis.

However, no beneficial effect of 25(OH)D on pre-DM was observed in either RCS or logistic regression, with the upper confidence interval exceeding 1, suggesting that the threshold of 35.02 ng/mL was not significant and presumably higher. Possible reasons for this are as follows. In the present study, we found no difference in 25(OH)D concentration distribution between the pre-DM and normal group, suggesting a similar condition of vitamin D deficiency. Since pre-DM is an intermediate state with glycemic status above the normal range but below the clinical T2DM [32], its mechanism is currently unclear. To date, insulin resistance and impaired incretin action are thought to be central to the pathophysiology of pre-DM and T2DM [1], whereas some experts hypothesize and speculate that calcium homeostasis, inflammatory responses and oxidative stress, as well as vitamin D-regulated levels of adipokines, such as leptin and lipocalin, are some of the possible mechanisms of progression to T2DM [33,34], and the potential causes will require future further exploration. In addition, in contrast to related studies, a valuable secondary analysis of a large-scale western D2d RCT trial showed that maintaining a 25(OH)D level in the range of 40~50 ng/mL reduced the risk of T2DM by 52% in pre-DM [35], and an IPD (individual participant data) analysis of the same study showed that compared to the reference group (20~29 ng/mL), the 25(OH)D concentration of ≥50 ng/mL group had a 76% lower risk of T2DM, and the 40~50 ng/mL group had a 62% lower risk of T2DM [36], suggesting that at least 40~50 ng/mL may have a preventive effect on pre-DM; this may be a plausible explanation for the unavailability of an effective threshold for pre-DM when the 25(OH)D concentration reached 35.02 ng/mL.

A stark reality is that the reported number of people with diabetes is just the tip of the iceberg. According to the IDF, nearly half of people with diabetes (both type 1 and type 2) living in low- and middle-income countries remain undiagnosed [37]. Of the potential T2DM parameters, such as FPG, HbA_1C_ and 2 h OGTT, which one will best predict T2DM in the coming years is a topic of discussion [38]. Furthermore, personalized interventions for different phenotypes will be a future trend [18]. In this study, we cautiously defined T2DM using three diagnostic criteria based on different glycemic traits and ultimately explored the 25(OH)D cut-off value of 29.06 ng/mL for three different phenotypes. In addition, when the plateau was reached in the LOESS curve, it showed that the 25(OH)D concentration was 28.95 ng/mL for FPG and 33.61 ng/mL for HbA_1C_, which was approximately close to or slightly above the 29.06 cut-off value of T2DM, validating our results.

Notably, vitamin D levels are also influenced by a variety of potential confounders [39], including age, season, ethnicity, education level, geographical factors, lifestyle, obesity and biological indicators reflecting metabolic status. All currently known relevant confounders were considered and adjusted during the fitting and validation process to ensure that an accurate optimal cut-off value was obtained.

The strengths of this study include its large sample size, its representative population of Chinese elderly and its use and validation of a variety of statistical methods to initially investigate the relationship between vitamin D and abnormal glycemic status. In addition, all the known relevant confounders were considered and adjusted to ensure the accuracy of the optimal thresholds. Notably, the use of three diagnostic criteria to validate the thresholds for different phenotypes is another strength of our findings. Exploring the relationship between vitamin D and T2DM for the elderly, a population at high risk for metabolic abnormalities, and obtaining the appropriate threshold, were other strengths of our study, which fills a gap in current research. It provides basic data for the development of PI-NCD values for the prevention of T2DM in the elderly.

Our study also has some limitations. First, reverse causality cannot be ruled out and not all confounders can be validated and corrected in cross-sectional studies; further causality evaluation studies, such as RCT and Mendelian randomization (MR) analyses, are needed. Second, the dose–response relationship and the sufficiency threshold for pre-DM have not been fully explored and need to be explored in further studies, with the underlying reasons to be elucidated. Finally, the true morphological and biological associations between serum vitamin D and T2DM risk may be more complex than those presented in our study.

## 5. Conclusions

In the present study, we found a nonlinear relationship between 25(OH)D concentration and the risk of abnormal glycemic status, and preliminarily explored 29.06 ng/mL as the optimal threshold, which is sufficient and beneficial for T2DM in Chinese older adults, but not for pre-DM. These findings provide basic data for relevant researchers to carry out further studies and provide basis for the development of PI-NCD values and the prevention of T2DM in the elderly.

## Figures and Tables

**Table 1:** Characteristics of Participants (*n* = 5856).

Parameters	*n*/Ratio (%)
Age group	
60~	1533 (26.18)
65~	1033 (17.64)
70~	947 (16.17)
75~	1511 (25.80)
80~	832 (14.21)
Gender	
Male	2976 (50.82)
Female	2880 (49.18)
Ethnicity	
Han	5231 (89.33)
Ethnic minorities	625 (10.67)
Education level	
Primary	4421 (75.50)
Medium	1279 (21.84)
Advanced	156 (2.66)
Region type	
Urban	3456 (59.02)
Rural	2400 (40.98)
District	
Eastern	2008 (34.29)
Central	1815 (30.99)
Western	2033 (34.72)
Season	
Spring	487 (8.32)
Autumn	3144 (53.69)
Winter	2225 (38.00)
Lifestyle	
Cigarette current/former smoker	2167 (37.00)
Alcohol consumer	1712 (29.23)
High intensity physical activity (Exercise)	119 (2.03)

The months of March, April and May are classified as spring; June, July and August as summer; September, October and November as autumn; and December, January and February as winter. High-intensity activity was defined as activities that require considerable physical effort or may cause a significant increase in respiration or heart rate, such as long-distance running, swimming, etc., for at least ten minutes.

**Table 2:** Clinical characteristics of different glycemic status groups.

Parameters	Total (*n* = 5856)	Median (*P*_25_−*P*_75_) of Parameters According to Different Glycemic Status Groups			*p* Value
		T2DM (*n* = 613)	Pre-DM (*n* = 1750)	Normal (*n* = 3493)	
Biochemistry					
25(OH)D (ng/mL)	18.62 (13.46~24.86)	17.31 (12.51~22.30) ^b^	18.90 (14.17~24.92) ^a^	18.73 (13.31~25.17) ^a^	<0.001
TC (mmol/L)	4.87 (4.23~5.55)	4.95 (4.27~5.75) ^a^	5.03 (4.40~5.70) ^a^	4.77 (4.16~5.45) ^b^	<0.001
TG (mmol/L)	1.21 (0.86~1.73)	1.49 (1.05~2.21) ^a^	1.32 (0.91~1.89) ^b^	1.11 (0.80~1.57) ^c^	<0.001
HDL-c (mmol/L)	1.28 (1.07~1.52)	1.18 (0.97~1.40) ^c^	1.27 (1.06~1.50) ^b^	1.31 (1.10~1.54) ^a^	<0.001
LDL-c (mmol/L)	3.02 (2.46~3.62)	3.16 (2.54~3.82) ^a^	3.15 (2.60~3.78) ^a^	2.92 (2.39~3.50) ^b^	<0.001
FPG (mmol/L)	5.33 (4.91~5.88)	8.00 (7.26~9.65) ^a^	5.88 (5.66~6.20) ^b^	5.04 (4.71~5.29) ^c^	<0.001
HbA_1C_ (mmol/mol)	32.24 (26.78~36.61)	48.63 (37.70~60.66) ^a^	34.43 (28.96~38.80) ^b^	30.05 (25.68~33.33) ^c^	<0.001
Hb (g/L)	148.10 (136.39~159.83)	150.56 (139.83~162.35) ^a^	149.02 (138.30~160.88) ^a^	146.93 (135.01~158.80) ^b^	<0.001
UA (μmol/L)	304.60 (254.93~365.38)	306.00 (256.90~368.65) ^ab^	316.00 (267.60~375.23) ^a^	298.10 (250.95~360.00) ^b^	<0.001
Anthropometry					
BMI (kg/m^2^)	23.32 (20.99~25.83)	25.05 (22.61~27.76) ^a^	24.00 (21.60~26.70) ^b^	22.69 (20.55~25.11) ^c^	<0.001
Waist (cm)	82.25 (75.05~89.50)	87.50 (80.18~95.00) ^a^	84.45 (76.57~91.05) ^b^	80.45 (74.00~87.50) ^c^	<0.001
SBP (mmHg)	145.67 (131.33~161.92)	150.67 (136.67~165.17) ^a^	147.50 (134.33~162.33) ^a^	143.33 (129.33~160.67) ^b^	<0.001
DBP (mmHg)	79.00 (71.33~86.67)	79.00 (71.00~87.00) ^ab^	79.67 (72.67~87.67) ^a^	78.67 (70.67~86.33) ^b^	0.015
Heart rate	75.33 (68.00~83.33)	78.33 (71.33~87.67) ^a^	76.67 (69.67~85.33) ^b^	74.00 (67.00~82.00) ^c^	<0.001

Comparison between different glycemic status groups using the letter marking method, with different letters indicating the statistical difference between groups and the same letter indicating non-statistical difference. Abbreviations: 25(OH)D, 25-hydroxyvitamin D; TC, total cholesterol; TG, triglycerides; HDL-c, high-density lipoprotein cholesterol; LDL-c, low-density lipoprotein cholesterol; FPG, fasting plasma glucose; HbA_1C_, glycated hemoglobin; Hb, hemoglobin; UA, uric acid; BMI, body mass index; SBP, systolic blood pressure; DBP, diastolic blood pressure. T2DM, type 2 diabetes mellitus; pre-DM, prediabetes; normal, normoglycemic individual.

**Table 3:** Odds ratios for T2DM according to vitamin D status based on different standards of diagnosis.

Index		25(OH)D Concentration ng/mL (*n* = 4106)										Per 1 ng/mL of 25(OH)D Increment		
		<12.00 (*n* = 829)	12.00~ (*n* = 1492)			20.00~29.06 (*n* = 1235)			≥29.06 (*n* = 550)					
			*β*	*OR* (95%*CI*)	*p* Value	*β*	*OR* (95%*CI*)	*p* Value	*β*	*OR* (95%*CI*)	*p* Value	*β*	*OR* (95%*CI*)	*p* Value
Fasting glucose type T2DM	Crude ^#^	ref	0.073	1.076 (0.847~1.368)	0.549	−0.185	0.831 (0.642~1.076)	0.160	−0.903	0.405 (0.274~0.601)	0.000	−0.029	0.972 (0.961~0.983)	0.000
	Model 1		0.004	1.004 (0.776~1.299)	0.976	−0.212	0.809 (0.603~1.085)	0.157	−0.797	0.451 (0.291~0.698)	0.000	−0.026	0.974 (0.960~0.988)	0.000
	Model 2		0.000	1.000 (0.772~1.294)	0.999	−0.209	0.811 (0.605~1.089)	0.163	−0.788	0.455 (0.294~0.704)	0.000	−0.026	0.974 (0.961~0.988)	0.000
	Model 3		0.049	1.050 (0.807~1.365)	0.717	−0.124	0.883 (0.655~1.192)	0.418	−0.658	0.518 (0.332~0.808)	0.004	−0.022	0.979 (0.965~0.993)	0.003
Abnormal HbA_1C_ type of T2DM	Crude ^#^	ref	0.192	1.211 (0.917~1.601)	0.178	−0.332	0.717 (0.524~0.982)	0.038	−0.909	0.403 (0.250~0.649)	0.000	−0.032	0.968 (0.955~0.982)	0.000
	Model 1		0.165	1.179 (0.872~1.594)	0.285	−0.339	0.713 (0.498~1.020)	0.064	−0.774	0.461 (0.271~0.784)	0.004	−0.029	0.971 (0.955~0.988)	0.001
	Model 2		0.167	1.182 (0.873~1.598)	0.279	−0.336	0.715 (0.499~1.024)	0.067	−0.758	0.469 (0.276~0.797)	0.005	−0.028	0.972 (0.955~0.989)	0.001
	Model 3		0.227	1.255 (0.924~1.706)	0.146	−0.221	0.801 (0.556~1.155)	0.235	−0.578	0.561 (0.327~0.961)	0.035	−0.022	0.978 (0.961~0.996)	0.014
Mixed type of T2DM	Crude ^#^	ref	0.041	1.042 (0.831~1.307)	0.722	−0.231	0.794 (0.622~1.014)	0.064	−0.714	0.490 (0.347~0.692)	0.000	−0.026	0.974 (0.964~0.985)	0.000
	Model 1		0.012	1.012 (0.792~1.293)	0.923	−0.208	0.813 (0.614~1.075)	0.146	−0.548	0.578 (0.391~0.855)	0.006	−0.021	0.979 (0.966~0.992)	0.002
	Model 2		0.009	1.009 (0.789~1.289)	0.945	−0.205	0.815 (0.616~1.079)	0.153	−0.539	0.583 (0.394~0.863)	0.007	−0.021	0.979 (0.967~0.992)	0.002
	Model 3		0.048	1.049 (0.818~1.345)	0.706	−0.138	0.871 (0.655~1.158)	0.342	−0.432	0.649 (0.436~0.967)	0.033	−0.017	0.983 (0.970~0.996)	0.012

^#^ Crude: unadjusted. Model 1 adjusted age, gender, ethnicity, education level, region type, district, season, altitude, latitude, BMI. Model 2: on the basis of Model 1, further adjusted physical activity, smoking and drinking. Model 3: on the basis of the Model 2, further adjusted TC, TG, HDL-c, UA. Abbreviations: *β*, beta coefficients; BMI, body mass index; TC, total cholesterol; TG, triglycerides; HDL-c, high-density lipoprotein cholesterol; UA, uric acid. (1) Fasting glucose type of T2DM: having FPG ≥ 7.0 mmol/L. (2) Abnormal HbA_1C_ type of T2DM: with HbA_1C_ ≥ 6.5% (≥48 mmol/mol). (3) Mixed type of T2DM: consistent with (1) or (2).

**Table 4:** Odds ratios for pre-DM according to vitamin D status based on different standards of diagnosis.

Index		25(OH)D Concentration ng/mL (*n* = 4106)										Per 1 ng/mL of 25(OH)D Increment		
		<12.00 (*n* = 993)	12.00~ (*n* = 1887)			20.00~35.02 (*n* = 2096)			≥35.02 (*n* = 267)					
			*β*	*OR* (95%*CI*)	*p* Value	*β*	*OR* (95%*CI*)	*p* Value	*β*	*OR* (95%*CI*)	*p* Value	*β*	*OR* (95%*CI*)	*p* Value
Fasting glucose type pre-DM	Crude ^#^	ref	0.292	1.339 (1.124~1.595)	0.001	0.234	1.264 (1.064~1.502)	0.008	0.033	1.034 (0.757~1.413)	0.834	0.002	1.002 (0.995~1.009)	0.516
	Model 1		0.243	1.274 (1.060~1.532)	0.010	0.194	1.215 (0.997~1.480)	0.054	0.082	1.085 (0.772~1.525)	0.637	0.002	1.002 (0.994~1.011)	0.558
	Model 2		0.232	1.261 (1.048~1.517)	0.014	0.187	1.206 (0.990~1.470)	0.063	0.076	1.079 (0.767~1.516)	0.663	0.002	1.002 (0.994~1.011)	0.599
	Model 3		0.224	1.251 (1.039~1.508)	0.018	0.194	1.214 (0.994~1.482)	0.058	0.050	1.052 (0.746~1.483)	0.774	0.002	1.002 (0.994~1.011)	0.583
Abnormal HbA_1C_ type of pre-DM	Crude ^#^	ref	−0.118	0.889 (0.698~1.132)	0.339	−0.145	0.865 (0.682~1.097)	0.233	−0.181	0.834 (0.536~1.298)	0.421	−0.005	0.995 (0.985~1.005)	0.334
	Model 1		−0.163	0.849 (0.658~1.095)	0.208	−0.184	0.832 (0.632~1.096)	0.190	−0.116	0.890 (0.550~1.441)	0.636	−0.004	0.996 (0.984~1.009)	0.574
	Model 2		−0.159	0.853 (0.661~1.101)	0.221	−0.180	0.835 (0.634~1.100)	0.200	−0.120	0.887 (0.548~1.437)	0.626	−0.004	0.996 (0.984~1.009)	0.568
	Model 3		−0.174	0.840 (0.650~1.087)	0.185	−0.186	0.830 (0.629~1.096)	0.189	−0.133	0.876 (0.539~1.422)	0.591	−0.002	0.998 (0.985~1.010)	0.736
Mixed type of pre-DM	Crude ^#^	ref	0.189	1.208 (1.024~1.425)	0.025	0.172	1.187 (1.009~1.397)	0.039	−0.021	0.979 (0.729~1.314)	0.887	0.002	1.002 (0.995~1.008)	0.614
	Model 1		0.137	1.147 (0.963~1.366)	0.124	0.119	1.126 (0.934~1.358)	0.214	0.003	1.003 (0.726~1.386)	0.984	0.001	1.001 (0.993~1.009)	0.771
	Model 2		0.134	1.143 (0.960~1.362)	0.134	0.117	1.124 (0.932~1.356)	0.220	−0.001	0.999 (0.723~1.380)	0.996	0.001	1.001 (0.993~1.009)	0.802
	Model 3		0.127	1.135 (0.951~1.354)	0.160	0.125	1.133 (0.937~1.370)	0.198	−0.024	0.977 (0.704~1.354)	0.887	0.001	1.001 (0.993~1.010)	0.722

^#^ Crude: unadjusted. Model 1 adjusted age, gender, ethnicity, education level, region type, district, season, altitude, latitude, BMI. Model 2: on the basis of Model 1, further adjusted physical activity, smoking and drinking. Model 3: on the basis of Model 2, further adjusted TC, TG, HDL-c, UA. Abbreviations: *β*, beta coefficients; BMI, body mass index; TC, total cholesterol; TG, triglycerides; HDL-c, high-density lipoprotein cholesterol; UA, uric acid. (1) Fasting glucose type pre-DM: FPG in the range of 5.6~6.9 mmol/L. (2) Abnormal HbA_1C_ type of pre-DM: HbA_1C_ in the range of 5.7~6.4% (39~47 mmol/mol). (3) Mixed type of pre-DM: consistent with (1) or (2).

## Data Availability

The dataset for the present study is available from the corresponding author upon reasonable request.

## Supplementary Materials

The following supporting information can be downloaded at: https://www.mdpi.com/article/10.3390/metabo14110579/s1, Table S1: Pearson correlation coefficient between variables; Table S2: Partial correlation coefficient of serum 25(OH)D and glycemic traits indicators by multiple linear regression.
