# Supplementary material for: Dose–Response Relationship Between Serum 25(OH)D and the Risk of Abnormal Glycemic Status in Chinese Older Adults
Source: Metabolites. 2024 Oct 26;14(11):579. doi: 10.3390/metabo14110579 (PMC11597016; doi:10.3390/metabo14110579)
Supplement: Supplementary file 1 [file metabolites-14-00579-s001.zip › metabolites-3258937-supplementary.pdf]

Supplementary Table 1. Pearson correlation coefficient between variables

variables	r /p value	VD	age	altitude	latitude	TC	TG	HDL-c	LDL-c	FPG	HbA_{1C}	Hb	UA	BMI	Waist	SBP	DBP
VD	*r*	1.000	-0.056**	-0.250**	-0.374**	0.014	-0.105**	0.083**	-0.007	-0.042**	-0.057**	-0.022	0.191**	-0.121**	-0.100**	-0.064**	-0.019
	p value		0.000	0.000	0.000	0.268	0.000	0.000	0.584	0.001	0.000	0.090	0.000	0.000	0.000	0.000	0.140
age	*r*	-0.056**	1.000	-0.031*	-0.041**	-0.068**	-0.054**	-0.028*	-0.071**	-0.002	-0.029*	-0.111**	0.087**	-0.142**	-0.070**	0.129**	-0.150**
	p value	0.000		0.019	0.002	0.000	0.000	0.031	0.000	0.874	0.029	0.000	0.000	0.000	0.000	0.000	0.000
altitude	*r*	-0.250**	-0.031*	1.000	0.032*	-0.076**	-0.017	-0.013	-0.077**	-0.102**	-0.040**	0.222**	-0.015	-0.058**	-0.038**	-0.016	0.052**
	p value	0.000	0.019		0.016	0.000	0.198	0.331	0.000	0.000	0.002	0.000	0.257	0.000	0.004	0.210	0.000
latitude	*r*	-0.374**	-0.041**	0.032*	1.000	-0.089**	0.065**	-0.118**	-0.060**	0.054**	0.042**	0.123**	-0.186**	0.214**	0.173**	0.047**	0.041**
	p value	0.000	0.002	0.016		0.000	0.000	0.000	0.000	0.000	0.001	0.000	0.000	0.000	0.000	0.000	0.002
TC	*r*	0.014	-0.068**	-0.076**	-0.089**	1.000	0.257**	0.311**	0.932**	0.059**	0.069**	0.030*	0.061**	0.091**	0.076**	0.075**	0.069**
	p value	0.268	0.000	0.000	0.000		0.000	0.000	0.000	0.000	0.000	0.024	0.000	0.000	0.000	0.000	0.000
TG	*r*	-0.105**	-0.054**	-0.017	0.065**	0.257**	1.000	-0.415**	0.284**	0.186**	0.142**	0.073**	0.168**	0.261**	0.240**	0.071**	0.086**
	p value	0.000	0.000	0.198	0.000	0.000		0.000	0.000	0.000	0.000	0.000	0.000	0.000	0.000	0.000	0.000
HDL-c	*r*	0.083**	-0.028*	-0.013	-0.118**	0.311**	-0.415**	1.000	0.041**	-0.106**	-0.107**	-0.058**	-0.118**	-0.301**	-0.285**	0.000	-0.013
	p value	0.000	0.031	0.331	0.000	0.000	0.000		0.002	0.000	0.000	0.000	0.000	0.000	0.000	0.978	0.304
LDL-c	*r*	-0.007	-0.071**	-0.077**	-0.060**	0.932**	0.284**	0.041**	1.000	0.081**	0.114**	0.053**	0.093**	0.192**	0.169**	0.070**	0.075**
	p value	0.584	0.000	0.000	0.000	0.000	0.000	0.002		0.000	0.000	0.000	0.000	0.000	0.000	0.000	0.000
FPG	*r*	-0.042**	-0.002	-0.102**	0.054**	0.059**	0.186**	-0.106**	0.081**	1.000	0.672**	0.050**	0.006	0.170**	0.160**	0.056**	0.022
	p value	0.001	0.874	0.000	0.000	0.000	0.000	0.000	0.000		0.000	0.000	0.632	0.000	0.000	0.000	0.093
HbA_{1C}	*r*	-0.057**	-0.029*	-0.040**	0.042**	0.069**	0.142**	-0.107**	0.114**	0.672**	1.000	0.071**	-0.016	0.184**	0.159**	0.045**	0.020
	p value	0.000	0.029	0.002	0.001	0.000	0.000	0.000	0.000	0.000		0.000	0.221	0.000	0.000	0.001	0.122
Hb	*r*	-0.022	-0.111**	0.222**	0.123**	0.030*	0.073**	-0.058**	0.053**	0.050**	0.071**	1.000	0.108**	0.118**	0.147**	0.002	0.171**
	p value	0.090	0.000	0.000	0.000	0.024	0.000	0.000	0.000	0.000	0.000		0.000	0.000	0.000	0.876	0.000
UA	*r*	0.191**	0.087**	-0.015	-0.186**	0.061**	0.168**	-0.118**	0.093**	0.006	-0.016	0.108**	1.000	0.141**	0.169**	0.004	0.055**
	p value	0.000	0.000	0.257	0.000	0.000	0.000	0.000	0.000	0.632	0.221	0.000		0.000	0.000	0.750	0.000

BMI	*r*	-0.121**	-0.142**	-0.058**	0.214**	0.091**	0.261**	-0.301**	0.192**	0.170**	0.184**	0.118**	0.141**	1.000	0.743**	0.160**	0.191**
	p value	0.000	0.000	0.000	0.000	0.000	0.000	0.000	0.000	0.000	0.000	0.000	0.000		0.000	0.000	0.000
Waist	*r*	-0.100**	-0.070**	-0.038**	0.173**	0.076**	0.240**	-0.285**	0.169**	0.160**	0.159**	0.147**	0.169**	0.743**	1.000	0.139**	0.170**
	p value	0.000	0.000	0.004	0.000	0.000	0.000	0.000	0.000	0.000	0.000	0.000	0.000	0.000		0.000	0.000
SBP	*r*	-0.064**	0.129**	-0.016	0.047**	0.075**	0.071**	0.000	0.070**	0.056**	0.045**	0.002	0.004	0.160**	0.139**	1.000	0.582**
	p value	0.000	0.000	0.210	0.000	0.000	0.000	0.978	0.000	0.000	0.001	0.876	0.750	0.000	0.000		0.000
DBP	*r*	-0.019	-0.150**	0.052**	0.041**	0.069**	0.086**	-0.013	0.075**	0.022	0.020	0.171**	0.055**	0.191**	0.170**	0.582**	1.000
	p value	0.140	0.000	0.000	0.002	0.000	0.000	0.304	0.000	0.093	0.122	0.000	0.000	0.000	0.000	0.000	

Note. Significance labeling: *$p \le 0.05$; **$p \le 0.01$. Abbreviation: 25(OH)D, 25-hydroxyvitamin D; TC, total cholesterol; TG, triglycerides; HDL-c, high-density lipoprotein cholesterol; LDL-c, low-density lipoprotein cholesterol; FPG, fasting plasma glucose; HbA_{1C}, glycated hemoglobin; Hb, hemoglobin; UA, uric acid; BMI, body mass index; SBP, systolic blood pressure; DBP, diastolic blood pressure. T2DM, type 2 diabetes mellitus; Pre-DM, prediabetes; Normal, normoglycemic individual.

Supplementary Table 2. Partial correlation coefficient of serum 25(OH)D and glycemic traits indicators by multiple linear regression

Variables	25(OH)D (ng/mL)		FPG (mmol/L)		HbA_{1C} (mmol/mol)	
	Partial Correlation Coefficient (95% *CI*)	*p* value	Partial Correlation Coefficient (95% *CI*)	*p* value	Partial Correlation Coefficient (95% *CI*)	*p* value
age*	-0.106	0.000	0.031	0.019	0.008	0.528
gender	-0.126	0.000	0.008	0.552	0.024	0.066
ethnicity	-0.006	0.670	-0.025	0.056	-0.052	0.000
education level	-0.066	0.000	0.019	0.152	0.021	0.110
region type	0.091	0.000	-0.036	0.006	-0.065	0.000
district	-0.083	0.000	-0.026	0.043	-0.036	0.005
season	-0.051	0.000	-0.017	0.193	-0.001	0.961
altitude*	-0.190	0.000	-0.056	0.000	0.008	0.549
latitude*	-0.339	0.000	0.003	0.841	-0.020	0.125
BMI*	-0.056	0.000	0.100	0.000	0.119	0.000
physical activity	0.002	0.860	0.004	0.744	-0.018	0.177
smoking	0.003	0.825	0.026	0.045	0.005	0.692
drinking	-0.049	0.000	-0.003	0.809	0.015	0.244
TC*	0.000	0.991	0.001	0.923	0.035	0.008
TG*	-0.064	0.000	0.123	0.000	0.064	0.000
HDL-c*	0.006	0.634	-0.006	0.673	-0.034	0.008
Hb*	0.027	0.037	0.054	0.000	0.061	0.000
UA*	0.109	0.000	-0.039	0.003	-0.054	0.000

Note. *indicates the numeric variables. LDL-c was excluded due to multicollinearity. Abbreviation: 25(OH)D, 25-hydroxyvitamin D; FPG, fasting plasma glucose; HbA1C, glycated hemoglobin; BMI, body mass index; TC, total cholesterol; TG, triglycerides; HDL-c, high-density lipoprotein cholesterol; LDL-c, low-density lipoprotein cholesterol; Hb, hemoglobin; UA, uric acid.
